# Overexpression of leptin receptor in human glioblastoma: Correlation with vasculogenic mimicry and poor prognosis

**DOI:** 10.18632/oncotarget.17344

**Published:** 2017-04-21

**Authors:** Guosheng Han, Yanan Li, Yiqun Cao, Zhijian Yue, Yuhui Zhang, Laixing Wang, Jianmin Liu

**Affiliations:** ^1^ Department of Neurosurgery, Changhai Hospital, Second Military Medical University, Shanghai, China

**Keywords:** glioblastoma, leptin receptor, vasculogenic mimicry, glial to mesenchymal transition, glioblastoma stem cells

## Abstract

Vasculogenic mimicry (VM) was an important tumor blood supply to complement the endothelial cell-dependent angiogenesis, while leptin and receptor (ObR) involved in angiogenesis in glioblastoma has been reported on previous study, but the relationship between ObR expression and VM formation in human glioblastoma tissues, as well as their prognostic significance still remains unclear. In our study, we found that VM recognized by CD31-/PAS+ immunohistochemical staining in glioblastoma tissues showed a positive correlation with leptin expression (*r* = 0.58, *P* < 0.01), as well as ObR expression in glioblastoma tissues (*r* = 0.61, *P* < 0.01). Association of glial to mesenchymal transition (GMT)-related molecular with ObR expression and VM formation in glioblastoma tissues indicated that ObR-positive glioblastoma cells with GMT phenotype might be more likely to constitute VM, and co-expression of ObR and CD133 or Nestin to constitute the channel impliated that ObR-positive glioblastoma cells displayed glioblastoma stem cells (GSC) properties. Moreover, Kaplan–Meier statistical analysis showed that patients with more VM or ObR expression displayed poorer prognosis for overall survival times than patients with less expression (VM^high^ vs. VM^low^: *P* = 0.033; ObR^high^ vs. ObR^low^: *P* = 0.009). And ObR+ glioblastoma cells with GSC characteristic were mostly involved in VM formation, whereas a little part of cells were also related to microvascular density (MVD), which suggested that ObR was an important target for anticancer therapy, so further related studies were needed to improve glioblastoma treatment.

## INTRODUCTION

Angiogenesis plays a significant role in tumor growth and metastasis, however, the unsatisfactory effects of anti-angiogenesis drugs on anticancer progression was indicated that there may be other blood supply forms in tumor tissues besides angiogenesis [1, 2]. Vasculogenic mimicry (VM), as a newly-defined pattern of tumor blood supply, provided a special vasculogenic-like networks to complement the endothelial-cell-dependent angiogenesis and vasculogenesis [3–5]. Maniotis *et al*. firstly reported the presence of these new tumor cells-dependent vessels in highly aggressive uveal melanomas [5], since then, this phenomenon has been confirmed successively in several malignant tumors, including glioblastoma [6]. VM has also been implicated in glioma metastasis and poor prognosis. Patients with glioma of type II microvascular pattern (MVP), including VM, have a poorer clinical outcome than do those of type I MVP, which is rich in microvascular sprouting (MS) and vascular cluster (VC) [7].

In recent years, epidemiological data suggest that obesity is associated with tumorigenesis and development. As product of obesity gene from adipocytes, leptin is defined as potent angiogenic factor involving in tumorigenesis, angiogenesis and metastasis [8–10], and the expression of leptin receptor (ObR) in malignant carcinoma was confirmed to be corresponded with tumor neoangiogenesis significantly [11]. Riolfi *et al*. previously demonstrated that the expression of leptin and ObR in human brain tumor tissues correlates with the degree of malignancy, and the highest levels of both markers are detected in glioblastoma multiform (GBM) [12]. Furthermore, leptin was observed to stimulate tube formation and enhance proliferation of endothelial cells directly, and the peptide ObR antagonist could inhibit these pro-angiogenic effects of leptin derived from glioma cells [13].

Although leptin and ObR involved in angiogenesis in glioma has been reported on previous study, the relationship between ObR expression and VM formation in human glioblastoma tissues and the relevance of their co-existence within glioblastoma invasion or prognostic significance remain unclear. The aims of our study were to examine the expression patterns of ObR and VM in human glioblastoma samples, and to further identify the correlation of ObR expression and VM, as well as their relevance to prognostic roles.

## RESULTS

### ObR expression and VM formation in human glioblastoma specimens

**Figure 1:**
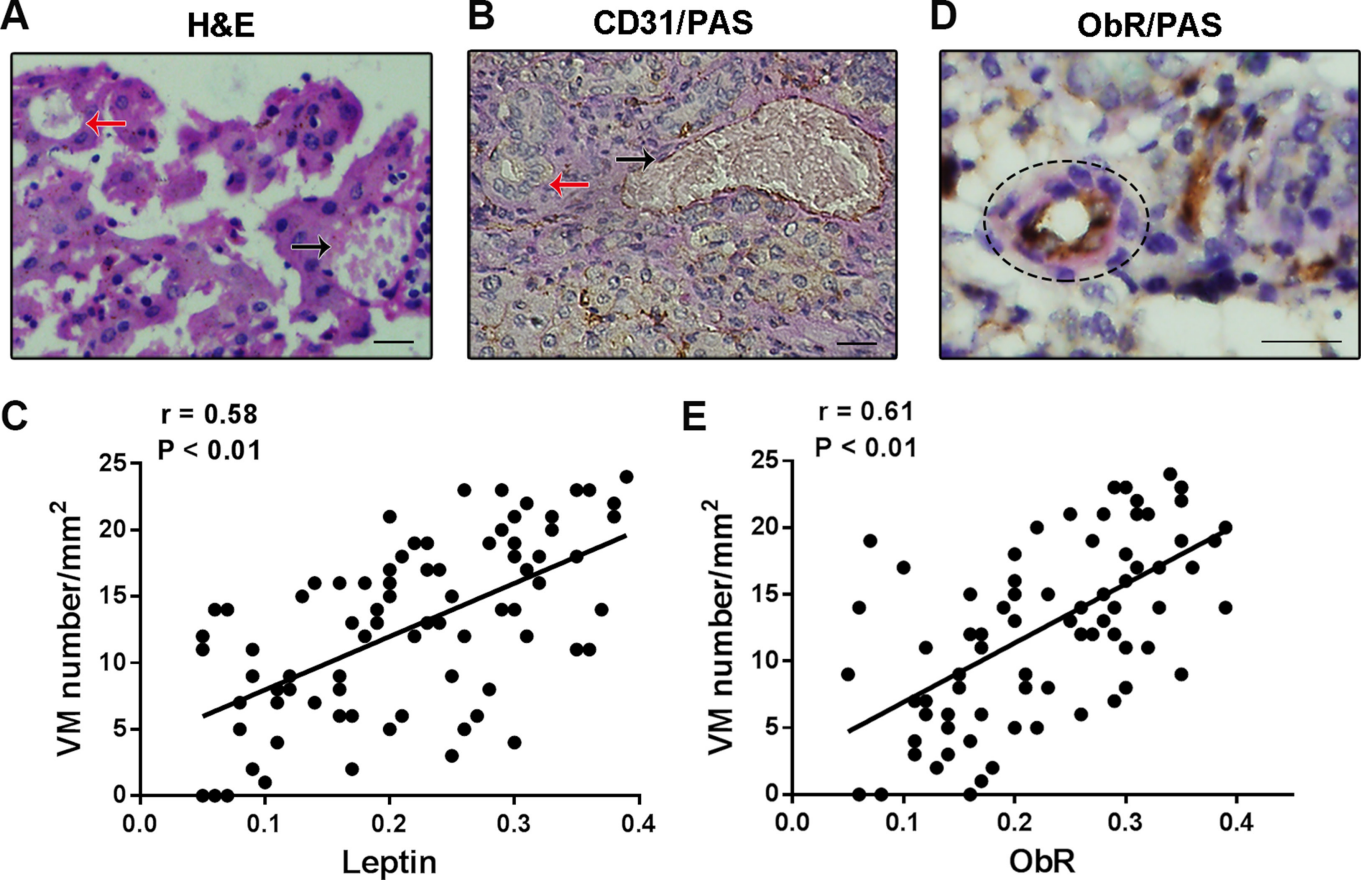
The correlation of ObR expression and VM formation in human glioblastoma specimens (**A**) H&E staining showed that the glioblastoma microcirculation system containing red blood cells was comprised of spindle endothelial cells (black arrows) as well as malignant tumor cells (red arrows); (**B**) CD31 and PAS double staining was used to identify VM and tumor blood vessels (red arrow indicates VM and black arrows indicates blood vessel); (**C**) VM formation was showed an positive correlation with leptin expression in glioblastoma tissues (*r* = 0.58, *P* < 0.01); (**D**) Several VMforming tumor cells presented high ObR expression detected by ObR/PAS double staining; (**E**) Spearman's rank test showed that ObR-positive expression was directly correlated with VM formation (*r* = 0.61, *P* < 0.01).

Glioblastoma blood vessels or VM can be identified based on H&E staining and histochemical double staining. Results of H&E staining showed that the glioblastoma microcirculation system containing red blood cells was comprised of spindle endothelial cells (Figure 1A, black arrows) as well as malignant tumor cells (Figure 1A, red arrows). Furthermore, CD31 and periodic acid-schiff (PAS) double staining was recruited to identify the endothelium in glioblastoma tissue sections and the basement membrane of tumor blood vessels. The spindle cells were positive for CD31 and the basement membrane was positive for PAS staining, however, the walls of VM channels made of glioblastoma cells were negative for CD31, but positive for PAS staining (Figure 1B, red arrow indicates VM, black arrows indicates blood vessel).

The serum concentration of leptin in human glioblastoma was always confirmed highly, but the correlation of leptin and VM in glioblastoma tissues was still unclear. We collected a cohort of 82 human glioblastoma tissues, which had been identified by pathological diagnosis. VM in glioblastoma tissues was recognized by CD31-/PAS+ immunohistochemical staining and leptin expression in tissues was measured by immunohistochemistry. As shown in Figure 1C, the statistical analysis indicated that a positive correlation was noted between the expression of leptin and VM in glioblastoma tissues (*r* = 0.58, *P* < 0.01). The expression of leptin receptor was further detected by immunohistochemistry in the glioblastoma tissues, and the positive signals of ObR were found to be located in the cytomembrane and cytoplasm. Notably, we found that several VMforming tumor cells presented high ObR expression detected by ObR/PAS double staining (Figure 1D). Of the 82 cases analyzed, 54 (65.9%) were positive for ObR or VM formation respectively. Among them, 45 were positive for both markers, 19 were both negative, 9 were ObR positive only, and 9 were VM positive only, and the statistical analysis revealed that ObR-positive expression is directly correlated with VM formation (Figure 1E, *r* = 0.61, *P* < 0.01).

### Association of GMT-related molecular with ObR expression and VM formation in human glioblastoma

**Figure 2:**
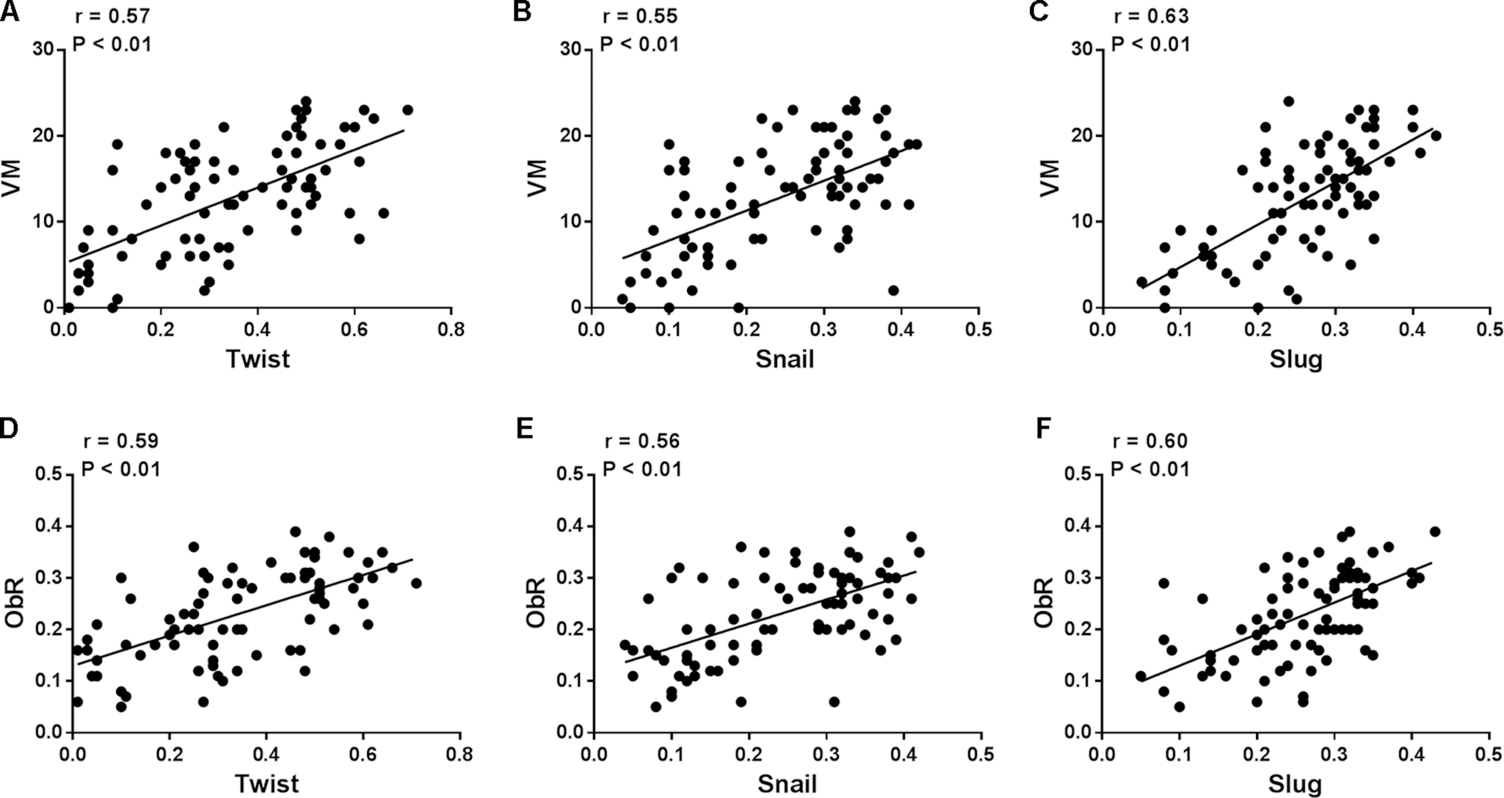
ObR expression or VM formation was associated with GMT-related molecular in human glioblastoma (**A**–**C**) the expression levels of GMT regulators including Twist, Snail, and Slug were increased in glioblastoma tissues with more VM (Twist: *r* = 0.57, *P* < 0.01; Snail: *r* = 0.55, *P* < 0.01; Slug: *r* = 0.63, *P* < 0.01); (**D**–**F**) Glioblastoma specimens that expressed high levels of GMT-related molecules had high ObR expression, whereas glioblastoma tissues with low or no GMT-related molecules expression had fewer evidence of ObR (Twist: *r* = 0.59, *P* < 0.01; Snail: *r* = 0.56, *P* < 0.01; Slug: *r* = 0.60, *P* < 0.01).

Recently, evidence has shown that epithelial-mesenchymal transition (EMT) is involved in the process of tumor VM formation [14], and our previous study has showed that leptin promoted glioblastoma cells invasion and metastasis [15], thus, we hypothesis that the association of leptin receptor expression and VM formation might be related to the EMT phenotype. Due to being developmentally derived from the neuroepithelial lineage of cells, Mahabir et al. employed the term “glial to mesenchymal transition (GMT)” instead of “EMT” in glioblastoma [16]. We detected the expression of GMT-related molecules in the glioblastoma tissues and found that VM formation was indeed associated with GMT phenotype. Specifically, the expression levels of GMT regulators including Twist, Snail, and Slug were increased in glioblastoma tissues with more VM (Figure 2A–2C. Twist: *r* = 0.67, *P* < 0.01; Snail: *r* = 0.56, *P* < 0.01; Slug: *r* = 0.63, *P* < 0.01). In addition, glioblastoma specimens that expressed high levels of GMT-related molecules had high ObR expression, whereas glioblastoma tissues with low or no GMT-related molecules expression had fewer evidence of ObR (Figure 2D–2F. Twist: *r* = 0.59, *P* < 0.01; Snail: *r* = 0.56, *P* < 0.01; Slug: *r* = 0.60, *P* < 0.01). Therefore, significant association was found between the existence of GMT-related molecular and VM formation, as well as ObR expression in human glioblastoma, which indicated that ObR-positive glioblastoma cells with GMT phenotype might be more likely to constitute VM.

### Relationship between GSC and ObR expression as well as VM formation in human glioblastoma

**Figure 3:**
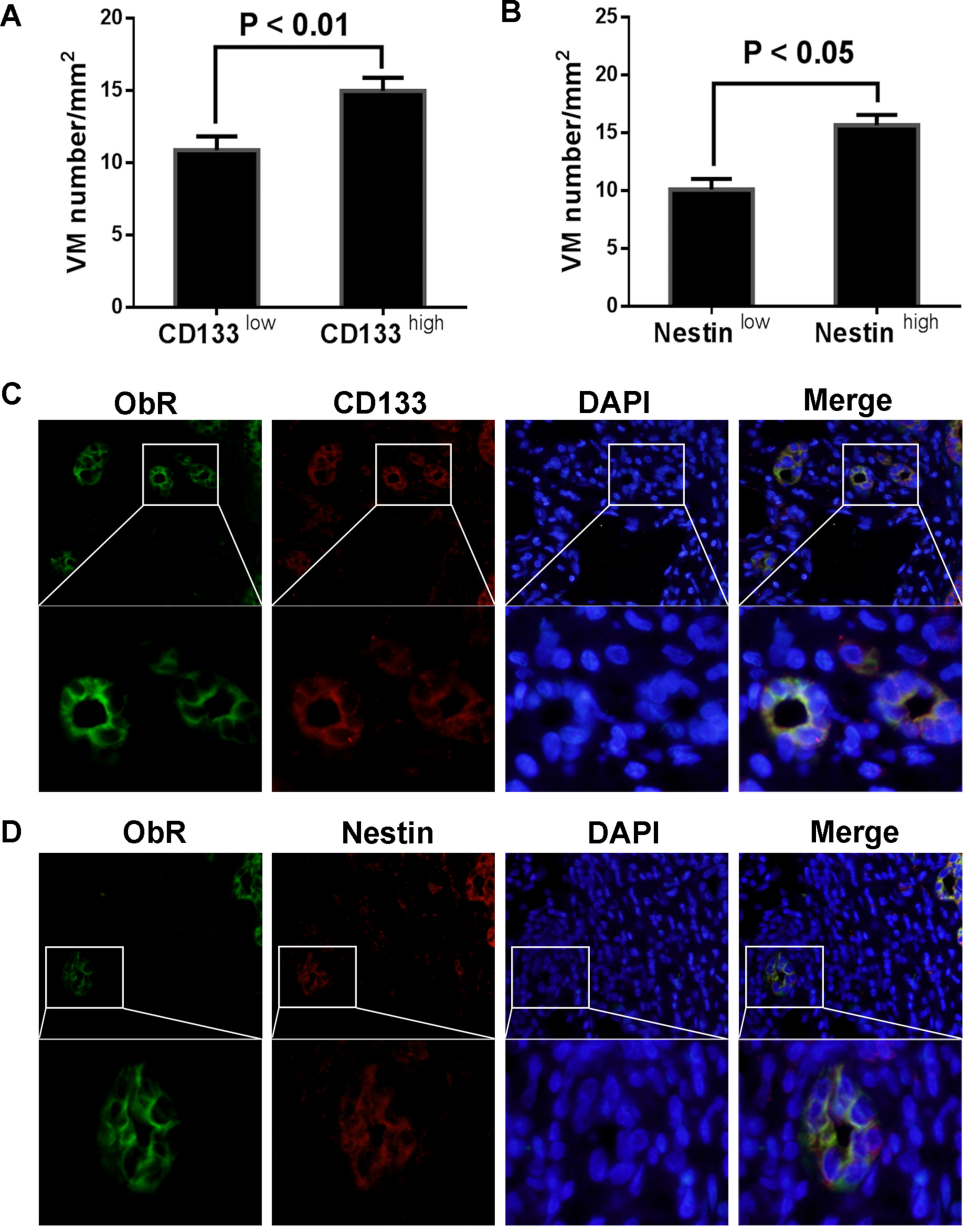
ObR+ glioblastoma cells with GSC markers were involved in VM formation (**A**, **B**) The glioblastoma tissues with high expression of CD133 or Nestin presented more VM formation; (**C**, **D**) Double immunofluorescent staining demonstrated that the co-expression of ObR and CD133 or Nestin to constitute the channel indicated that ObR-positive glioblastoma cells displayed GSC properties.

EMT has been confirmed to be associated with the acquisition of CSC properties in tumor. Plausibly, GSCs in glioblastoma might be implicated in VM formation by induction of GMT. In our study, the expression of GSCs markers, CD133 and Nestin, has also been detected in glioblastoma tissues by immunohistochemistry. The statistical analysis revealed that those tissues with high expression of CD133 or Nestin presented more VM formation (Figure 3A, 3B), which confirmed the previous researches about GSC attributes being able to form vascularlike structures. We also used double immunofluorescent staining to demonstrate the association between the expression of ObR and CD133, as well as Nestin, in glioblastoma tissues. In order to differentiate GSCs from endothelium cells, we confirmed these CD133 or Nestin-positive cells with no CD31 expression (Supplementary Figure 1). As shown in Figure 3C and 3D, the co-expression of ObR and CD133 or Nestin to constitute the channel indicated that ObR-positive glioblastoma cells displayed GSC properties. Furthermore, we performed some experiments about VM formation *in vitro*, the method of which was used as described [17]. And we found that the expression of ObR was higher in CD133+ U87 glioblastoma cells (GSCs) compared with CD133– cells, if we deleted ObR expression in GSCs, VM formation was decreased correspondingly, the results of which were showed in Supplementary Figure 2. These data implicated that ObR expression might be important in VM formation through stemness maintenance and GMT induction.

### Prognostic significance of ObR expression and VM formation in human glioblastoma

**Figure 4:**
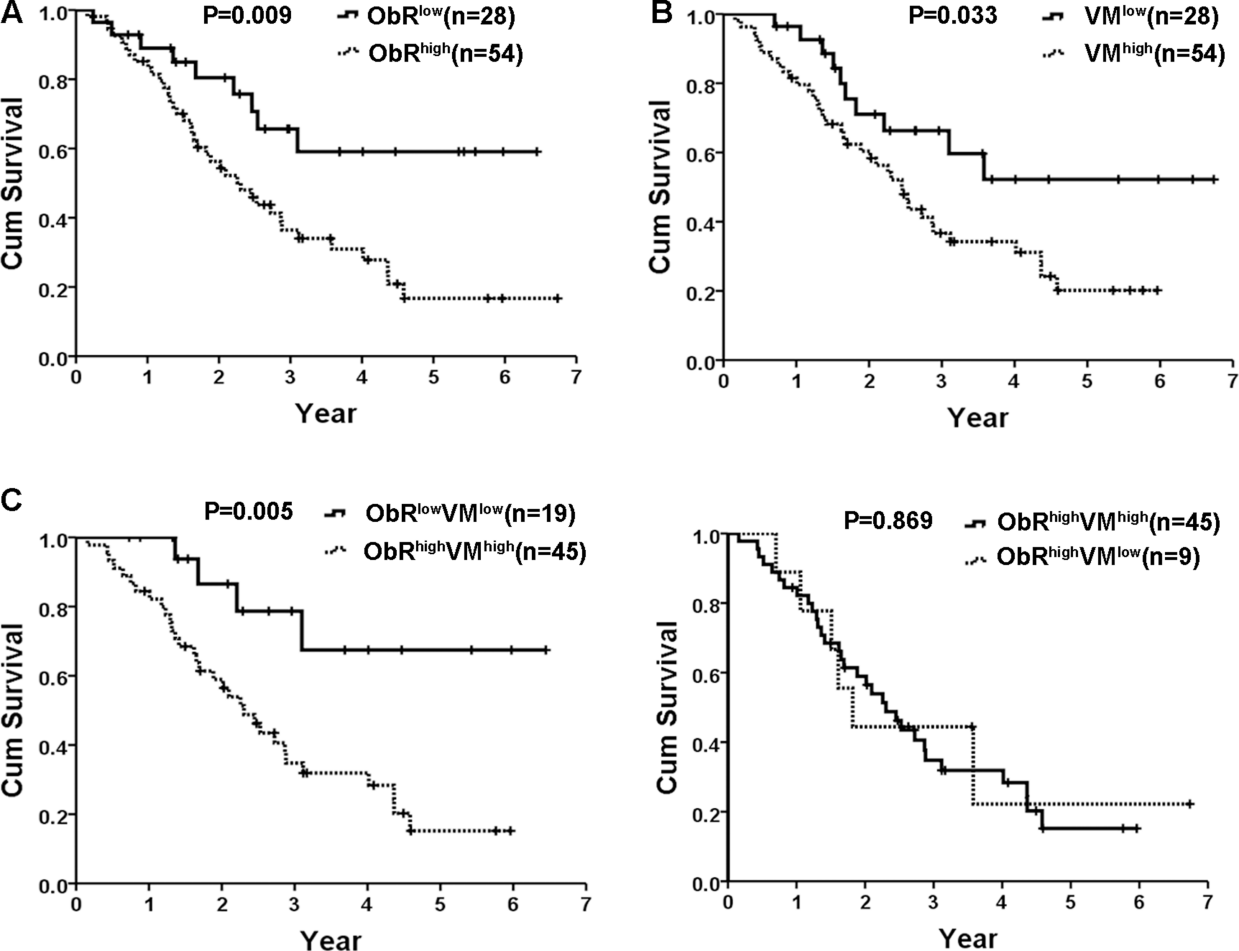
Kaplan–Meier statistical analysis showed prognostic significance of ObR expression and VM formation in human glioblastoma (**A**) The overall survival period of glioblastoma patients with ObR high expression was enormously shorter than that of glioblastoma patients with ObR low expression (*P* = 0.009); (**B**) Patients with high VM displayed poorer prognosis for overall survival times than patients with low VM (*P* = 0.033); (**C**) Patients with both high expression of ObR and VM formation exhibited worse survival time when compared with ObR^low^VM^low^ group (*P* = 0.005); (**D**) No significant difference was found between patients with ObR^high^VM^high^ and ObR^high^VM^low^ for overall survival time (*P* = 0.869).

To verify the clinical significance of ObR and VM, all 82 glioblastoma patients were followed up and the relationship between their outcomes and ObR expression as well as VM was examined. Kaplan–Meier statistical analysis showed that the mean overall survival period of ObR low expression group was 4.560 ± 0.499 years, whereas that of ObR high expression group was only 2.923 ± 0.307 years, and the overall survival period of glioblastoma patients with ObR high expression was enormously shorter than that of glioblastoma patients with ObR low expression (Figure 4A, *P* = 0.009). Consistently, patients with high VM displayed poorer prognosis for overall survival times than patients with low VM. The mean overall survival time were 2.878 (2.331–3.426) years for patients with high VM, whereas the corresponding median overall survival time were 4.504 (3.459–5.548) years for patients with low VM (Figure 4B, *P* = 0.033).

**Figure 5:**
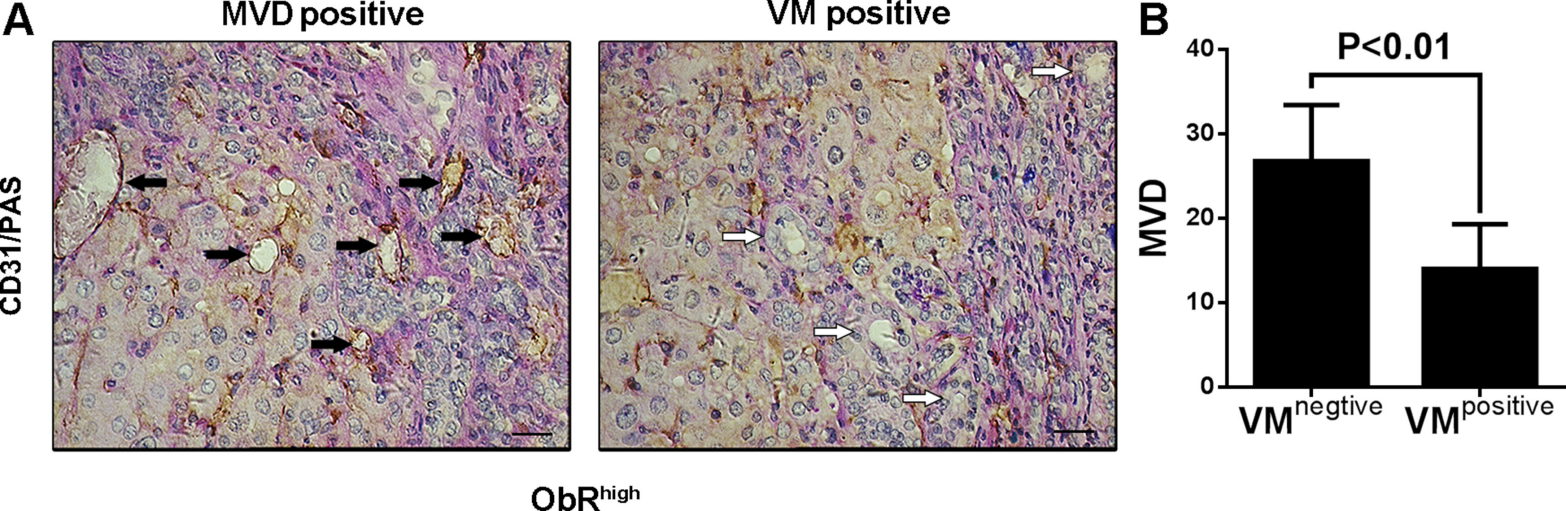
ObR+ glioblastoma cells with GSC characteristic were mostly involved in VM formation, whereas a little part of cells were also related to MVD (**A**) CD31 and PAS were stained to calculate MVD (CD31+/PAS+) and VM (CD31–/PAS+) (black arrow indicates MVD, white arrows indicates VM). (**B**) Of all 57 glioblastoma tissues with ObR overexpression, the levels of MVD in VM-negative tissues were 26.78 ± 2.22, which was higher than that in VM-positive tissues (14.02 ± 0.79, *P* < 0.01).

Notably, patients with both high expression of ObR and VM formation exhibited worse survival time when compared with ObR^low^VM^low^ group (*P* = 0.005, Figure 4C). However, no significant difference was found between patients with ObR^high^VM^high^ and ObR^high^VM^low^ for overall survival time (*P* = 0.869, Figure 4D). In reality, ObR overexpression not only related to VM formation but also related to glioblastoma angiogenesis. We then explored potential association between VM and Microvascular density (MVD) in glioblastoma tissues with ObR overexpression. CD31 and PAS were stained to calculate MVD (CD31+/PAS+) and VM (CD31-/PAS+) (Figure 5A, black arrow indicates MVD, white arrows indicates VM). Of all 54 glioblastoma tissues with ObR overexpression, the levels of MVD in VM-negative tissues were 26.78 ± 2.22, which was higher than that in VM-positive tissues (14.02 ± 0.79, *P* < 0.01, Figure 5B), which indicated that VM and MVD were complementary glioblastoma blood supplies, and ObR+ glioblastoma cells with GSC characteristic were mostly involved in VM formation, whereas a little part of cells were also related to MVD.

## DISCUSSION

As one of the most vascularized tumors, the increased microvasculature is a major hallmark in GBM pathology [18, 19]. Many factors have been reported to be involved in glioblastoma angiogenesis. Felar et al. have demonstrated that human GBM cells in culture produce biologically active leptin that can induce growth and pro-angiogenic effects in endothelial cells [13], which confirmed the role for obesity in creating a microenvironment favorable for glioblastoma angiogenesis. Previous studies of tumor vascularization mainly focused on the angiogenesis that was established by endothelial cells. With the discovery of VM, the channels were lined by tumor cells instead of endothelial cells, several studies successively found this phenomenon could be another main source of tumor blood supply. Additionally, tumors exhibiting VM were usually related to more aggressive biology and poor prognosis. Nevertheless, whether the pro-angiogenic effect of leptin and ObR including VM formation was still unclear.

In this study, expression of blood vessels and VM was investigated by immunohistochemistry in human glioblastoma tissues. The results showed that VM was existed to constitute glioblastoma microcirculation system, which was in accordance with the previous report [7, 20, 21]. Chen *et al*. had demonstrated that VM might act as a complement to ensure glioblastoma blood supply, especially in regions with less microvessel density [2]. In addition, VM was found to be more frequently expressed with higher expression of ObR in glioblastoma tissues and higher leptin in patients’ serum, and the co-expression of VM with ObR was further confirmed the importance of leptin in VM formation. The significant association was observed VM channels in tumor correlated with increasing malignancy and higher aggressiveness, suggesting that EMT could contribute to VM formation. Sun *et al*. had reported that Twist1 was frequently overexpressed in the nuclear relocation occurring in VM-positive HCCs [22]. As an important EMT regulator, the aberrant expression of ZEB1 was also found in VM forming cancer cell lines [23]. In addition, hypoxia microenvironment enhanced tumor VM formation mainly through increased expression of EMT regulators [24, 25]. As shown in our results, the expression of GMT regulators including Twist, Snail, and Slug was increased in glioblastoma tissues with more VM, which was consistent with the previous *in vitro* studies. Moreover, there was increasing evidence suggesting a wider biological role for leptin/ObR related to tumor cell motility and invasiveness, and our data indicated that ObR-positive glioblastoma cells had a higher metastasis rate than did those with negative ObR expression, the association between ObR and GMT-related molecules expression in glioblastoma tissues was further confirmed the capabilities of ObR on tumor motility and invasion. Therefore, the expression of ObR in tumor cell to consist the channel maybe imply that ObR promotes glioblastoma cells VM formation, perhaps through inducing glioblastoma cells GMT phenotype.

As a process about regaining dedifferentiated phenotypes and mesenchymal features, EMT induction acquired tumor cells with CSCs characteristic, this dedifferentiation process might partly become a new mechanism for CSCs origin [1, 26, 27]. Whereas the EMT transcription factors were inhibited in CSCs, the cells would lose CSCs properties and increase apoptosis [28]. Tumor cells capable of VM formation exhibited high plasticity, and more evidence indicated that CSCs had the capacity of transdifferentiation and contribute to VM in tumor [14]. Our previous research indicated that ObR-positive glioblastoma cells possessed GSCs characteristic, such as self-renewal, invasive ability, and chemoresistance property [29], which may be considered as a bridge between GMT and VM formation. In glioblastoma tissues, we confirmed that VM numbers was correlated with GSC markers expression, such as CD133, Nestin. Furthermore, the coexpression of ObR and CD133, as well as Nestin, indicated the ObR-positive glioblastoma cells were GSCs. Therefore, the involvement of ObR+ glioblastoma cells into VM formation might be due to GMT induction and GSCs characteristic acquirement.

In recent years, a number of studies has analyzed the relationship between the markers of GSCs and prognosis of patients with glioblastoma. Wu *et al*. conducted a systematic review and meta-analysis to evaluate the association between CD133 or Nestin expression and the outcome of glioblastoma patients, and they found that high CDl33 expression was an independent risk factor for glioblastoma patients’ prognosis, especially WHO IV glioblastomas, and high Nestin expression was an independent risk factor for glioblastoma patients’ prognosis with grade WHO II–III [30]. Due to GSCs with high expression ObR, the high expression ObR would have an impact on the outcome of glioblastoma patients. In this study, our findings were consistent with the above mentioned results, that patients with ObR high expression in tumors had a shorter survival time than those with ObR low expression. In addition, ObR positive glioblastoma cells consisted VM was shown to be correlated with patient clinical outcome, which indicated that glioblastoma patients with more VM tended towards tumor metastasis and had a lower survival rate [2, 31, 32]. ObR overexpression was not only related to VM formation but also related to glioblastoma angiogenesis with low VM formation, therefore, there was no significant difference between patients with ObR^high^VM^high^ and ObR^high^VM^low^ for overall survival time. VM and MVD were complementary glioblastoma blood supplies, and ObR positive glioblastoma cells with GSC characteristic were mostly involved in VM formation, whereas a little part of cells were also related to MVD, which suggested that ObR was an important target for anticancer therapy, so further related studies were needed to improve glioblastoma treatment.

## MATERIALS AND METHODS

### Patients and clinical data

82 archived, formalin-fixed, paraffin-embedded glioblastoma tissue specimens were all acquired from Changhai Hospital between January 1996 and December 2006. The glioblastoma tumor grade of patients was diagnosed histologically according to the WHO classification. There were 48 men and 34 women, the age range was 34–77 years and the mean ages were 56 and 62 year respectively. The survival time was measured from the date of surgery to the date of death. Total period of follow up was 8–84 months. The present study was approved by the Hospital Research Ethics Committee and performed in accordance with institutional and state guidelines on the use of human tissue specimens for experimental purposes. Written informed consent was obtained from all patients.

### Immunohistochemical and periodic acid-schiff (PAS) double-staining

Tissue sections (4–5 μm) were deparaffinized and dehydrated using a graded series of ethanol solutions and stained with hematoxylin-eosin (H&E) as standard procedures, and the serial sections were stained with double staining of CD31 or ObR and PAS. Endogeneous peroxidase was then inactivated with 3% hydrogen peroxide at room temperature for 20 minutes. Then the slides were soaked in 0.1 mol/L citrate buffer (pH 6.0) and placed in an autoclave at 121°C for 2 minutes for antigen retrieval. After washing with PBS (pH 7.4), the sections were blocked with 1% BSA diluted in PBS at 37°C for 30 minutes, and then incubated with anti-CD31 protein IgG (1:50, Abcam, Cambridge, UK) or anti-ObR protein IgG (1:50; Dako, Glostrup, Denmark) at 4°C overnight, after being rinsed with PBS again, the sections were incubated with HRP-conjugated goat anti-mouse/rabbit antibody and DAB (DAKO, Glostrup, Denmark) washing distilled water, then the section were incubated with 0.5% PAS for 10 min in a dark chamber and washing with distilled water for 3 min. Finally all of these sections were counterstained with hematoxylin.

All sections with immunohistochemical staining were observed and the pictures were photographed by an Olympus microscope (IX-70 OLYMPUS, Japan). Four representative fields within each section were randomly chosen and captured under 200X. The integrated optical density (IOD) in each image was measured with the same setting for all the slides, and the density was calculated as IOD/total area of each image.

### Double-fluorescence immunostaining

Double-fluorescence immunostaining of the glioblastoma tumor tissue was performed with a sequential fluorescent method as described [33]. The primary antibodies against ObR and CD133 were all from Abcam, and Alexa488-conjugated goat anti-mouse IgG and Alexa568-conjugated goat anti-rabbit IgG (all from Invitrogen) were used as secondary antibodies. Immunofluorescence was observed with the Olympus IX-71.

### Statistical analysis

Spearman's rank test was used to analyze the correlation between ObR expression and VM formation, as well as the correlation between ObR expression or VM formation with GMT markers. Survival curves were estimated using Kaplan–Meier method and compared by log rank test. All statistical analyses were performed using the SPSS software system (version 20.0; SPSS, Chicago, IL, USA). *P* < 0.05 was considered statistically significant.

## SUPPLEMENTARY MATERIALS FIGURES


