# Supplementary material for: Overexpression of leptin receptor in human glioblastoma: Correlation with vasculogenic mimicry and poor prognosis
Source: Oncotarget. 2017 Apr 21;8(35):58163–71. doi: 10.18632/oncotarget.17344 (PMC5601641; doi:10.18632/oncotarget.17344)
Supplement: Supplementary file 1 [file oncotarget-08-58163-s001.pdf]

Overexpression of leptin receptor in human glioblastoma: Correlation with vasculogenic mimicry and poor prognosis

Supplementary Materials

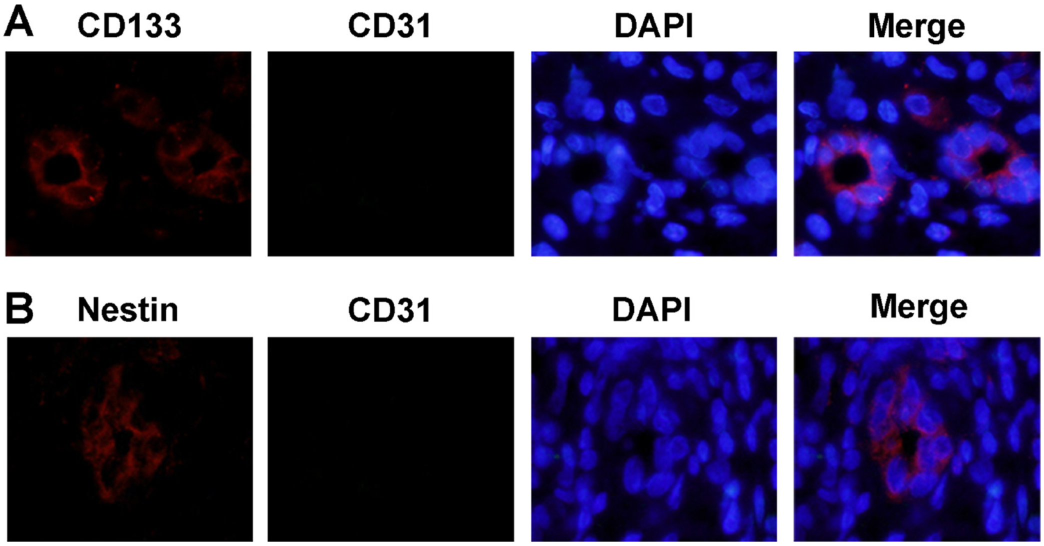

Supplementary Figure 1: The expression of CD31 and CD133 or Nestin in glioblastoma tissues. CD133 (**A**) or Nestin (**B**)-positive cells had no expression of CD31, which indicated that these cells just were GSCs, but not endothelium cells.

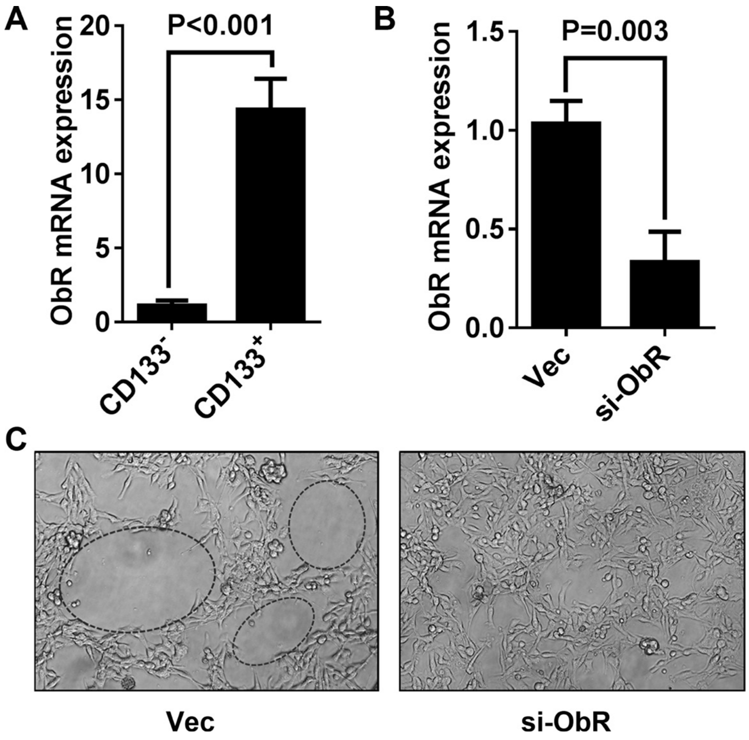

Supplementary Figure 2: The experiment about relationship of ObR expression and VM formation *in vitro*. (**A**) The expression of ObR was higher in CD133+ U87 glioblastoma cells (GSCs) compared with CD133– cells; siRNA was used to delete ObR expression in CD133+ GSCs (**B**), and VM formation was decreased correspondingly (**C**).
